# Manganese in Drinking Water and Intellectual Impairment in School-Age Children

**DOI:** 10.1289/ehp.1103485

**Published:** 2011-06

**Authors:** Hong Chen, Ray Copes

**Affiliations:** Ontario Agency for Health Protection and Promotion, Toronto, Ontario, Canada, E-mail: hong.chen@ohapp.ca

We read with interest the the article by Bouchard et al. (2011) on the effect of manganese in drinking water on children’s IQ (intelligence quotient). In this cross-sectional study, the authors examined IQ scores in relation to manganese exposure using four exposure metrics: *a*) concentration of manganese in tap water; *b*) concentration of manganese in hair samples; *c*) estimate of manganese intake from water consumption; and *e*) estimate of manganese intake from diet consumption.

One key finding from the study of Bouchard et al. (2011) is that a higher concentration of manganese in tap water was significantly associated with lower IQ. Compared with the other three exposure metrics used in the study, the concentration of manganese in water followed an almost perfect dose–response relationship with children’s IQ, and it was shown to be a better predictor of lower IQ than the exposure metrics. We found this surprising for three reasons. First, in their analysis of the association between concentration of manganese in tap water and IQ, Bouchard et al. included the entire study population (*n* = 362). We consider this inappropriate because 33% of the study participants (*n* = 121) did not drink tap water at home. Thus, these 121 children may have experienced much lower exposure to manganese from tap water than the remaining children in the study. Second, if we consider the highest quintile of water-manganese concentration (median, 216 μg/L), the estimated manganese intake from water would be ≤ 0.43 mg/day for half of the children in this exposure group, assuming a daily water intake of 2 L. Even at this level, the intake of manganese from water was still far below the daily intake recommended by the Institute of Medicine (2001): children 1–3 years of age (1.2 mg/day) and children 4–13 years of age (1.5–1.9 mg/day). Third, Bouchard et al. reported that the children’s manganese intake from food was more than two orders of magnitude compared to the amount ingested from water. This suggests that if elevated manganese was causally related to lower IQ, the decrease in IQ was more likely due to the intake of manganese from both water and food sources than from water alone. While one can postulate differences in bioavailability between manganese in food and in water, these would need to be considerable to result in equal or greater uptake from water than from food.

The utility of hair as a biomarker for human exposure to manganese has yet not been established [Agency for Toxic Substances and Disease Registry (ATSDR) 2001]. There is still a lack of standard procedure for collection of hair samples as well as insufficient evidence to demonstrate the effect of washing hair on analytical results (ATSDR 2001). Bouchard et al. (2011) excluded children with dyed hair, but it would be interesting to also distinguish children with natural hair of different colors in the analysis, because levels of manganese in hair can vary by natural colors of hair.

Bouchard et al. (2011) generated an interesting hypothesis on neurotoxicity of water manganese in children at a level that is currently considered to have no adverse effect (World Health Organization 2008), but we believe more studies will be needed to confirm their findings. To better characterize human exposure to manganese from water, it is important for future studies to quantify bioavailability of manganese from water and from food sources. In addition, employing a prospective study design and controlling for all possible risk factors—including overall nutritional status—will be critical. Additionally, comparing hair with other biomarkers of manganese exposure would be another area to explore for future studies.
